# Universal Pretreatment Development for Low-input Proteomics Using Lauryl Maltose Neopentyl Glycol

**DOI:** 10.1016/j.mcpro.2024.100745

**Published:** 2024-03-04

**Authors:** Ryo Konno, Masaki Ishikawa, Daisuke Nakajima, Yusuke Endo, Osamu Ohara, Yusuke Kawashima

**Affiliations:** 1 Department of Applied Genomics, Kazusa DNA Research Institute, Kisarazu, Chiba, Japan; 2 Department of Frontier Research and Development, Kazusa DNA Research Institute, Kisarazu, Chiba, Japan; 3 Graduate School of Science, Kitasato University, Sagamihara, Kanagawa, Japan

**Keywords:** DMNG, LMNG, peptide loss, SCP, SP3

## Abstract

In recent years, there has been a growing demand for low-input proteomics, particularly in the context of single-cell proteomics (SCP). In this study, we have developed a lauryl maltose neopentyl glycol (LMNG)-assisted sample preparation (LASP) method. This method effectively reduces protein and peptide loss in samples by incorporating LMNG, a surfactant, into the digestion solution and subsequently removing the LMNG simply *via* reversed phase solid-phase extraction. The advantage of removing LMNG during sample preparation for general proteomic analysis is the prevention of mass spectrometry (MS) contamination. When we applied the LASP method to the low-input SP3 method and on-bead digestion in coimmunoprecipitation-MS, we observed a significant improvement in the recovery of the digested peptides. Furthermore, we have established a simple and easy sample preparation method for SCP based on the LASP method and identified a median of 1175 proteins from a single HEK239F cell using liquid chromatography (LC)-MS/MS with a throughput of 80 samples per day.

## Highlights

• Developing low-input proteomics method.
• This method involves using LMNG surfactant for reducing protein and peptide loss.
• Removing the LMNG simply via reversed phase solid-phase extraction.
• Identifying a median of 1175 proteins from a single cell (80 samples/day).

## Graphical Abstract

**Figure:**
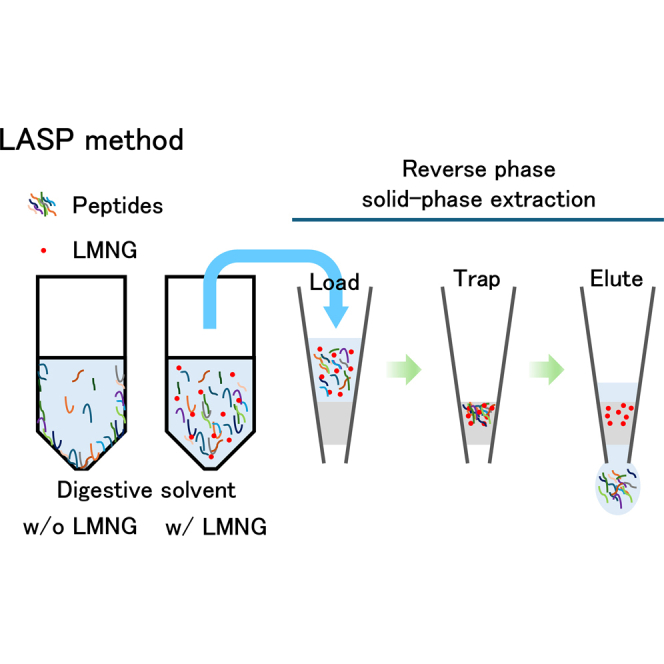

## In Brief

We developed a lauryl maltose neopentyl glycol (LMNG)-assisted sample preparation (LASP) method for low-input proteomics. LMNG surfactant is added for reducing protein and peptide loss into the digestion solution, then easily removed the LMNG via reversed-phase solid-phase-extraction. Peptide recovery significantly improved upon SP3 and coimmunoprecipitation-MS employing LASP method. Using the LASP method for single-cell proteomics, it successfully identified a median of 1175 proteins from a single HEK239F cell upon using LC-MS/MS with a throughput of 80 samples per day.

In samples with sufficient cell numbers for proteomic analysis, the field has reached an era where a single-shot measurement can reveal more than 10,000 proteins (1, 2, 3). Proteomic analysis is expected to make significant contributions to the fields of biology and medicine. The next major challenge in proteomic analysis is the active development of techniques for low-input samples such as single-cell proteomics (SCP) and spatial proteome analysis. However, as the amount of protein in the sample decreases, the rates of protein and peptide loss increase, rendering single-cell and spatial proteomic analyses even more challenging. Techniques have been developed for sample preparation in nanoliter-scale liquid volumes, such as nanoPOTS (4, 5, 6) and the Oile-Air-Droplet method (7), which can minimize nonspecific adsorption for low-input samples. However, these methods are not universally applicable and require special equipment. Therefore, it is necessary to prevent protein and peptide loss using a simple method that is as versatile as possible, without the requirement for specialized equipment.

The major sample preparation steps for proteomic analysis include the following: (1) protein extraction, (2) reduction-alkylation, (3) enzymatic digestion, (4) desalting using a reversed phase-solid-phase extraction (RP-SPE) column, (5) drying, and (6) redissolution. Additionally, with the advent of the SP3 (8, 9) and S-Trap methods (10), versatile methods for removing surfactants and salts from lysates and enzymatic digestion have become widely adopted. However, in low-input samples, proteins and peptides are lost during long incubations, such as during protein digestion, and upon transferring samples to a different tube. The use of surfactants is desirable to reduce these losses. However, surfactants are trapped in the RP-SPE column, elute with peptides, and cannot be easily removed. Surfactants known not to interfere with peptide analysis by liquid chromatography-mass spectrometry (LC-MS/MS) include *n*-dodecyl-β-D-maltoside (DDM) (11, 12, 13). Yet, in the case of low-input samples, it is a common practice to dry the eluted sample from an RP-SPE column, dissolve the peptides in a small amount of solvent, and subsequently analyze the concentrated peptides by LC-MS/MS. This method naturally increases the risk of MS contamination, as the concentrated surfactant is analyzed with concentrated peptides. Depending on the surfactant type, methods to remove the surfactant after digestion by precipitation (14), phase transfer surfactants (15), or surfactant decomposition (16, 17) have been developed. However, these treatments increase the number of work steps, resulting in various disadvantages, including increased labor, reduced reproducibility, and peptide loss. Therefore, it is ideal to remove surfactants by a general process without any special requirements.

In this study, our objective was to identify surfactants that can be effectively removed using RP-SPE columns without any special treatment. Since it would be difficult to find a surfactant that does not adsorb onto an RP-SPE column, as surfactants have both hydrophilic and hydrophobic groups, we attempted to find a surfactant with strong affinity to an RP-SPE column and to identify conditions where only peptides elute without eluting the surfactant. The surfactants, including DDM, were sourced from sugar-type surfactants, which fall under a category known for longer elution times compared to peptides. A total of 12 surfactants were examined in this study. In addition, we explored the use of low concentrations of surfactants for peptide redissolution aiming to reduce peptide loss during the drying step after RP-SPE. Ultimately, we established a simple and easy pretreatment method for SCP and performed high-throughput single-cell proteomics, processing up to 80 samples per day, using Evosep One.

## Experimental Procedures

### LC-MS Measurement of Surfactants

The surfactants (Table 1) were directly injected onto a 75 μm × 12 cm nanoLC column (Nikkyo Technos Co, Ltd) at 50 °C and then separated with a 30-min gradient (A = 0.1% formic acid (FA) in water, B = 0.1% FA in 80% acetonitrile [ACN]) consisting of 0 min 5% B, 25 min 95% B, 30 min 95% B at a flow rate of 300 nl/min using an UltiMate 3000 RSLCnano LC system (Thermo Fisher Scientific). The elution from the column was analyzed using a Q Exactive HF-X (Thermo Fisher Scientific) with data-dependent acquisition (DDA). MS1 spectra were collected in the range of 100 to 1500 m/z at a 60,000 resolution to set an auto-gain control (AGC) target of 1 × 10^6^ and a maximum injection time of 119 ms. The MS chromatograms of the monoisotopic mass (single charge) of each surfactant were obtained.

**Table 1:** Surfactant list

Sugar-based surfactant	Abbreviation	Molecular weight	Company	CAT#
*n*-Dodecyl-β-D-maltoside	DDM	510.62	FUJIFILM Wako Pure Chemical Corp	347-06163
*n*-Undecyl-β-D-maltoside	UDM	496.59	Merck	94206
*n*-Decyl-β-D-maltoside	DeDM	482.57	FUJIFILM Wako Pure Chemical Corp	349-08041
*n*-Octyl-β-D-maltoside	ODM	454.51	FUJIFILM Wako Pure Chemical Corp	340-90283
*n*-Nonyl-β-D-thiomaltoside	NDT	484.6	FUJIFILM Wako Pure Chemical Corp	343-06861
*n*-Octyl-β-D-glucoside	ODG	292.37	FUJIFILM Wako Pure Chemical Corp	346-05033
*n*-Heptyl-β-D-thioglucoside	HDT	294.41	FUJIFILM Wako Pure Chemical Corp	346-05371
*n*-Octyl-β-D-thioglucoside	ODT	308.44	FUJIFILM Wako Pure Chemical Corp	349-05361
Decyl maltose neopentyl glycol	DMNG	949.08	Anatrace	NG322
Lauryl maltose neopentyl glycol	LMNG	1005.19	Anatrace	NG310
Octyl glucose neopentyl glycol	OGNG	568.69	Anatrace	NG311
Trehalose C12	TC12	524.6	FUJIFILM Wako Pure Chemical Corp	340-09051

### The Tryptic Activity on the Different Concentrations of LMNG

Trypsin activity for different lauryl maltose neopentyl glycol (LMNG) concentrations was assessed by UV absorbance measurement using a Trypsin activity assay kits (CAT# ab102531, Abcam). LMNG concentrations in the digestion solutions were prepared at 0%, 0.01%, 0.02%, 0.04%, 0.08%, and 0.16%, and the solutions were supplemented with trypsin at a concentration of 6.25 mU. The trypsin activity was then examined using Multiskan sky TCD (Thermo Fisher Scientific), with absorbance at 405 nm measured every 20 min for up to 2 h and every hour after 2 h.

### Elution Conditions for SDB-STAGE Tip of LMNG, DMNG, and DDM

The SDB-STAGE tip (CAT# 7820-11200, GL Sciences Inc) was washed with 25 μl of 80% ACN in 0.1% TFA, followed by equilibration using 50 μl of 3% ACN in 0.1% TFA. Then, 10 μl of 0.001% LMNG, decyl maltose neopentyl glycol (DMNG), or DDM was loaded onto the tip, washed using 80 μl of 3% ACN in 0.1% TFA, and eluted stepwise with 50 μl of 30% ACN in 0.1% TFA, 40% ACN in 0.1% TFA, and 50% ACN in 0.1% TFA. An additional LMNG sample was eluted with 50 μl of 30% ACN in 0.1% TFA, 32% ACN in 0.1% TFA, 34% ACN in 0.1% TFA, 36% ACN in 0.1% TFA, 38% ACN in 0.1% TFA, and 40% ACN in 0.1% TFA, respectively. The eluate was dried using a centrifugal evaporator (miVac Duo Concentrator; Genevac Ltd). The dried sample was redissolved in 200 μl of distilled water and transferred to a normal vial (CAT# C5000-97, Thermo Fisher Scientific).

### Dried Peptide Mixtures

A total of 100 μg of K562 cell tryptic digest (CAT# V6951, Promega) was adjusted to 50 ng/μl with 50% ACN containing 0.1% TFA and dispensed in 10 μl each into normal tubes (1.5-ml safe-lock tube, CAT# 0030120086, Eppendorf), normal polypropylene (PP) vials (CAT# C5000-97, Thermo Fisher Scientific), or hydrophilic vials (ProteoSave vial, CAT# 11-19-1021-10, AMR Inc). These samples were dried in a centrifugal evaporator (miVac Duo concentrator) and redissolved in 25 μl of distilled water, 2% ACN containing 0.1% TFA, or 0.00125 to 0.04% surfactants in distilled water (Table 1).

### Protein Extraction From HEK293T Cells

HEK293T cells were cultured in a 10-cm dish at 80% confluence in Dulbecco's modified Eagle’s medium (DMEM) (Fujifilm Wako) containing 10% fetal bovine serum (Thermo Fisher Scientific) and 1% penicillin/streptomycin (Fujifilm Wako) at 37 °C in a 5% CO_2_ incubator. Proteins from the HEK293T cells were extracted in 100 mM Tris–HCl (pH 8.0) and 20 mM NaCl containing 4% SDS by sonication in Bioruptor II (Cosmo Bio) for 10 min. Protein concentration in the protein extract was determined using a Bicinchoninic acid protein assay kit (CAT# 23225, Thermo Fisher Scientific) and adjusted to 500 ng, 5 or 0.5 ng/μl with 100 mM Tris–HCl (pH 8.0), and 20 mM NaCl containing 4% SDS.

### Purification of Extracellular Vesicle (EVs) From Human Serum

Human serum EVs were purified using the Tim4-phosphatidylserine (PS) affinity method combined with the MagCapture Exosome Isolation Kit PS Ver.2 (Wako Pure Chemical) and Maelstrom 8 Autostage (Taiwan Advanced Nanotech Inc). Briefly, 150 μl of pooled healthy human serum was centrifuged at 3000*g* at 4 °C for 20 min, and then, 100 μl of supernatants were collected in different tubes. Subsequently, 300 μl of Tris-buffered saline and 1 μl of exosome binding enhancer (provided with the kit) were added to the supernatants and gently mixed. To prepare the beads for Tim4-PS affinity method, 30 μl of exosome capture beads (provided with the kit) was washed once with 250 μl of exosome immobilizing/washing buffer (provided with the kit). Then, the beads were mixed in 10 μl of biotin-labeled exosome capture (diluted by 250 μl of exosome immobilizing/washing buffer) and agitated at 1000 rpm for 10 min. After washing twice with 250 μl of exosome immobilizing/washing buffer, the beads were added to the samples. To bind EVs to the beads, the mixture was agitated at 1000 rpm for 2 h, followed by washing thrice with 250 μl of exosome immobilizing/washing buffer. Finally, bead-captured EVs were eluted with 100 mM Tris–HCl (pH 8.0) and 20 mM NaCl containing 4% SDS.

### Protein Digestion

The protein lysate, purified EVs, and biotinylated bovine serum albumin (BSA) (CAT# A8549-10MG; Sigma-Aldrich) were treated with 20 mM tris(2-carboxyethyl) phosphine at 80 °C for 10 min and alkylated using 35 mM iodoacetamide at room temperature for 30 min while being protected from light and subjected to clean up and digestion with SP3 using Maelstrom 8 Autostage. Briefly, two types of Sera-Mag SpeedBead carboxylate-modified magnetic particles (hydrophilic particles: CAT# 45152105050250 and hydrophobic particles: CAT# 65152105050250; Cytiva) were used. The beads were combined in a 1:1 (v/v) ratio, washed twice with distilled water, and reconstituted in distilled water at a concentration of 8 μg solids/μl. Subsequently, 20 μl of reconstituted beads were added to 200 μl of alkylated protein sample, followed by 99.5% ethyl alcohol, to reach the final concentration of 75% (v/v), with mixing for 5 min. The supernatant was discarded, and the pellet was washed twice with 80% ethyl alcohol. The beads were then resuspended in 80 μl of 50 mM Tris–HCl (pH 8.0) or 50 mM Tris–HCl (pH 8.0) containing 0.02% LMNG, followed by 500 ng of trypsin/Lys-C Mix (CAT# V5072, Promega) and mixed gently at 37 °C overnight to digest the proteins. The digested sample was acidified with 20 μl of 5% TFA and then sonicated with Bioruptor II (Cosmo Bio) at a high level for 5 min at room temperature. Samples were desalted using an SDB-STAGE tip or Evotip Pure (CAT# EV2015; Evosep Biosystems). The SDB-STAGE tip was washed with 25 μl of 80% ACN in 0.1% TFA, followed by equilibration using 50 μl of 3% ACN in 0.1% TFA. Then, the sample was loaded onto tip, washed using 80 μl of 3% ACN in 0.1% TFA, and eluted with 50 μl of 50% ACN in 0.1% TFA or 36% ACN in 0.1% TFA. The eluate was then dried using a centrifugal evaporator (miVac Duo Concentrator). The dried sample was redissolved in 8 μl of 2% ACN containing 0.1% TFA or 0.01% DMNG and transferred to the normal vial. Four microliters of the sample were injected into the LC-MS/MS system. Evotip Pure was used according to the manufacturer’s protocol. In brief, the tip was washed with 20 μl of ACN in 0.1% FA, followed by equilibration using 20 μl of 0.1% FA in water. Then, the sample was loaded onto the tip and washed using 20 μl of 0.1% FA in water. The tip was filled with 0.1% FA in water until LC−MS/MS measurements.

### CoIP and On-Bead Digestion

HeLa cells were cultured in a 10-cm dish at 80% confluence in DMEM (Fujifilm Wako) containing 10% fetal bovine serum (Thermo Fisher Scientific) and 1% penicillin/streptomycin (FUJIFILM Wako) at 37 °C in a 5% CO_2_ incubator. Immunoprecipitation (IP) lysis buffer (Pierce IP Lysis Buffer (CAT# 87788, Thermo Fisher Scientific) containing protease (CAT# 5892791001, cOmplete ULTRA Tablets, Sigma-Aldrich) and phosphatase inhibitors (CAT# 4906837001, PhosSTOP Tablets, Sigma-Aldrich) was added to HeLa cells and mixed at 4 °C for 30 min. The cell lysate was then centrifuged at 18,000*g* at 4 °C for 30 min, and the supernatant was collected. Protein concentration in the protein extract was determined using a Bicinchoninic acid protein assay kit (CAT# 23225, Thermo Fisher Scientific) and adjusted to 1 μg/μl with the IP lysis buffer containing protease and phosphatase inhibitors. Co-immunoprecipitation (coIP) of the HeLa cell lysate was performed using an automated Maelstrom 8 Autostage. The coIP experiments utilized Sera-Mag SpeedBeads Protein A/G Magnetic Particles (CAT# 17152104010150, Cytiva) anti-RELA (NF-κB p65) antibodies (CAT# ab16502, Abcam), and control (Rabbit immunoglobulin G, polyclonal) antibodies (CAT# ab171870, Abcam). To prepare the beads for IP, 1.5 μl of bead slurry in Tris buffered saline containing 0.05% Tween 20 (TBST) and 1% BSA (Blocker BSA, CAT# 37520, Thermo Fisher Scientific) was washed once with 500 μl of TBST containing 1% BSA. Then, the anti-RELA or control antibody was captured on the beads by mixing them with 3 μg anti-RELA or control antibody at room temperature for 30 min in 200 μl of TBST containing 1% BSA and washed twice in 500 μl of TBST to remove the unbound antibody. Subsequently, 200 μl of HeLa cell lysate (1 μg/μl) were added to the beads and incubated for 60 min at room temperature with gentle mixing, followed by washing once with 500 μl of the IP lysis buffer containing protease and phosphatase inhibitors, three times with 500 μl of TBST, and once with 500 μl of 50 mM Tris–HCl (pH 8.0). The beads were treated with 100 μl of 50 mM Tris–HCl (pH 8.0) or 50 mM Tris–HCl (pH 8.0) containing 0.02% LMNG, followed by 500 ng of Trypsine Platinum (CAT# VA9000, Promega), and mixed gently at 37 °C overnight to digest proteins. The beads were then aggregated from the digested sample using a magnetic stand (EpiMag HT (96-Well) Magnetic Separator; EpiGentek) to collect the supernatant. The collected sample was alkylated using 10 mM tris (2-carboxyethyl) phosphine and 40 mM 2-chloroacetamide at 80 °C for 15 min. Subsequently, the alkylated sample below room temperature was acidified with 20 μl of 5% TFA (total volume 130 μl) and desalted using SDB-STAGE tip (elution: 36% ACN in 0.1% TFA), followed by drying in a centrifugal evaporator (miVac Duo Concentrator). The dried sample was redissolved in 10 μl of 2% ACN containing 0.1% TFA or 0.01% DMNG and transferred to normal vials (Thermo Fisher Scientific). One microliter of each sample was injected into the LC-MS/MS system. The coIP experiments were performed with four biological replicates.

### Sample Preparation for SCP

As a collection plate for fluorescence-activated cell sorting (FACS) cells, 10 μl of 2 ng/μl trypsin/Lys-C Mix (CAT# V5072, Promega) in 0.3% acetic acid were added to a 96-well plate (CAT# 0030128664, Eppendorf), sealed with plate seal (CAT# 5010-21951, GL Sciences Inc) and stocked at −80 °C.

FreeStyle HEK293-F cells were cultured in DMEM medium supplemented with 10% fetal calf serum until they reached 80% confluence. Cells were collected, pelleted by centrifugation, and washed thrice with PBS. For FACS, 5 μl 4′,6-diamidino-2-phenylindole was added to the 5 ml single-cell solution, and cell sorting was performed on the 4′,6-diamidino-2-phenylindole-negative live cell population with BD FACSMelody cell sorter. Single or ten cells were sorted into the collection plates, brought to room temperature, sealed with plate seal (CAT# 5010-21951, GL Sciences Inc), and frozen at 80 °C until use. Single or ten cells in 96-well plates were brought to room temperature and centrifuged at 500*g* for 3 min. Five microliters of 500 mM Tris–HCl pH 8.0 with 0.08% LMNG or 500 mM Tris–HCl pH 8.0 were added to the plate, subjected to water bath-type sonication (Bioruptor II, Cosmo Bio) for 5 min, and mixed gently at 37 °C overnight to digest proteins. The plates were centrifuged at 500*g* for 3 min, and 5 μl of 5% TFA were added and then treated with Evotip Pure according to the manufacturer’s protocol.

### DDA-MS by Typical LC-MS/MS

The redissolved peptides were directly injected onto a 75 μm × 12 cm nanoLC nano-capillary column (Nikkyo Technos Co, Ltd) at 50 °C and then separated with a 30-min gradient (A = 0.1% FA in water, B = 0.1% FA in 80% ACN) consisting of 0 min 5% B, 30 min 45% B at a flow rate of 300 nl/min using an UltiMate 3000 RSLCnano LC system. The peptides eluted from the column were analyzed on an Orbitrap Exploris 480 (Thermo Fisher Scientific) using the InSpIon system (AMR) (18). MS1 spectra were collected in the range of 380 to 1240 m/z at a 60,000 resolution to set an AGC target of 3 × 10^6^ and a maximum injection time of 100 ms. The 50 most intense ions with charge states of 2+ to 5+ that exceeded 8.0 × 10^3^ were fragmented by collision induced dissociation with a normalized collision energy of 28%. MS2 spectra were collected in the range of more than 200 m/z at a 15,000 resolution to set an AGC target of 1 × 10^5^ and a maximum injection time of “Auto.” The dynamic exclusion time was set to 30 s.

### DIA-MS by Typical nanoLC-MS/MS

The redissolved peptides were directly injected onto a 75 μm × 12 cm nanoLC column (Nikkyo Technos Co, Ltd) at 50 °C and then separated with a 60-min gradient (A = 0.1% FA in water, B = 0.1% FA in 80% ACN) consisting of 0 min 8% B, 50 min 35% B, 57 min 70% B, 60 min 70% B at a flow rate of 200 nl/min using an UltiMate 3000 RSLCnano LC system. The peptides eluted from the column were analyzed using an Orbitrap Exploris 480 with an InSpIon system. MS1 spectra were collected in the range of 495 to 905 m/z at a 15,000 resolution to set an AGC target of 3 × 10^6^ and a maximum injection time of 23 ms. MS2 spectra were collected at more than 200 m/z at a 30,000 resolution to set an AGC target of 3 × 10^6^, a maximum injection time of “Auto,” and a normalized collision energy of 26%. The isolation width for MS2 was set to 8 m/z, and for the 500 to 900 m/z window pattern, an optimized window arrangement was used in Xcalibur 4.4 (Thermo Fisher Scientific).

### Evosep One LC-MS/MS

The Evotip Pure sample was analyzed using an Evosep One system (EVOSEP) equipped with an Orbitrap Exploris 480 mass spectrometer and InSpIon system. Evosep One was acquired using the Whisper 80 SPD method (gradient running time, 15 min; flow rate, 100 nl/min). The digested peptides were separated using an Aurora Rapid 75 C18 capillary column (5 cm × 75 μm i.d., particle size 1.7 μm; IonOpticks) at 60 °C. Mobile phases A and B consisted of 0.1% FA in H_2_O and 0.1% FA in ACN, respectively. The peptides eluted from the column were analyzed using an Orbitrap Exploris 480 instrument with data-independent acquisition (DIA). DIA parameters were slightly modified from a previously reported high-throughput proteomic analysis (19). MS1 spectra were collected in the range of 645 to 775 m/z at a 7500 resolution to set an AGC target of 1 × 10^6^ and a maximum injection time of 10 ms. MS2 spectra were collected at 200 to 1800 m/z at a 30,000 resolution to set an AGC target of 3 × 10^6^, a maximum injection time of “Auto,” and a normalized collision energy of 28%. The isolation width for MS2 was set to 8 m/z, and for the 650 to 770 m/z window pattern, an optimized window arrangement was used in Xcalibur 4.4 (Thermo Fisher Scientific).

To optimize DIA using the Whisper 80SPD method for smaller samples, we investigated the proteomic coverage of the 49 DIA methods using 1 ng of cell digest. These DIA methods were designed using different combinations of precursor mass ranges (m/z 120, 180, and 240), mass resolutions (30,000, 45,000, and 60,000), and isolation window widths (8, 12, 15, 20, and 24 Da). In all methods, MS1 parameters were set as follows: mass resolution, 7500; AGC target, 1 × 10^6^; and maximum injection time, 10 ms. In DIA, the maximum injection time values at mass resolutions of 30,000, 45,000, and 60,000 were set at 55, 87, and 119 ms, respectively. AGC target for MS2 was set to 3 × 10^6^ at 28% normalized collision energy.

### Protein Identification and Quantitative Analysis From MS Data

DDA-MS files were searched against the human protein sequence UniProt database (proteome ID UP000005640, 20,591 entries, downloaded on March 7, 2023) using Proteome Discoverer 3.0, with Sequest HT and Percolator (Thermo Fisher Scientific). The parameters were set as follows: enzyme, trypsin; maximum missed cleavage sites, 2; precursor mass tolerance, 10 ppm; fragment mass tolerance, 0.02 Da; static modification, cysteine carbamidomethylation; and dynamic modification, methionine oxidation. DIA-MS files were also searched against an *in silico* human spectral library using DIA-NN (version: 1.8.1, https://github.com/vdemichev/DiaNN) (20). First, a spectral library was generated from the human protein sequence UniProt database using DIA-NN. The Parameters for generating the spectral library were as follows: digestion enzyme, trypsin; missed cleavages, 1; peptide length range, 7 to 45; precursor charge range, 2 to 4; precursor m/z range, 395 to 1005; and fragment ion m/z range, 200 to 1800. “FASTA digest for library-free search/library generation;” “deep learning-based spectra, RTs, and IMs prediction;” “n-term M excision;” and “C carbamidomethylation” were enabled. The DIA-NN search parameters were as follows: mass accuracy, 10 ppm (15 ppm with Evosep One); MS1 accuracy, 10 ppm (15 ppm with Evosep One); protein inference, genes; neural network classifies, single-pass mode; quantification strategy, robust LC (high precision); and Cross-run normalization, off. “Unrelated runs,” “use isotopologues,” “heuristic protein inference,” and “no shared spectra” were enabled. The match between runs was turned off when searching for the identification of proteins and precursors and turned on for quantitative analysis. The protein identification threshold was set at 1% or less for both precursor and protein false discovery rates (FDRs).

### Phosphoproteomics

Hundred micrograms of peptide digest was dissolved in 80% ACN in 0.1% TFA. Dissolved peptides were added to Fe-NTA Magnetic Agarose (80 μl of suspension used; Cat# A52284, Thermo Fisher Scientific) washed with 80% ACN in 0.1% TFA and mixed for 60 min at room temperature, followed by washing twice with 1 ml of the 80% ACN in 0.1% TFA and once with 1 ml of 0.1% TFA. The beads were treated with 200 μl of 3% polyphosphoric acid in 0.1% TFA or 3% polyphosphoric acid in 0.1% TFA containing 0.02% LMNG and then mixed for 10 min at room temperature to elute the phosphopeptides. The eluted phosphopeptides were desalted by SDB-STAGE tip (elution: 36% ACN in 0.1% TFA). The dried sample was redissolved in 10 μl of 0.01% DMNG and transferred to a normal vial. The redissolved peptides were directly injected onto a 75 μm × 30 cm nanoLC column (CAT# HEB07503001718IWF, CoAnn Technologies) at 60 °C and then separated with a 100-min gradient (A = 0.1% FA in water, B = 0.1% FA in 80% ACN) consisting of 0 min 5% B, 86 min 35% B, 93 min 70% B, and 100 min 70% B at a flow rate of 150 nl/min using an UltiMate 3000 RSLCnano LC system. The peptides eluted from the column were analyzed on an Orbitrap Exploris 480 using the InSpIon system. MS1 spectra were collected in the range of 400 to 1500 m/z at a 60,000 resolution to set an AGC target of 3 × 10^6^ and a maximum injection time of “Auto.” The 50 most intense ions with charge states of 2+ to 4+ that exceeded 2.0 × 10^4^ were fragmented by collision induced dissociation with stepped normalized collision energies of 22%, 26%, and 30%. MS2 spectra were collected in the range of 200 to 1800 m/z at a 30,000 resolution to set an AGC target of 5 × 10^5^ and a maximum injection time of “Auto.” The dynamic exclusion time was set to 30 s. The MS files were searched against the human protein sequence UniProt database (proteome ID UP000005640, 20,591 entries, downloaded on March 7, 2023) using Proteome Discoverer 3.0 with Sequest HT and Percolator (Thermo Fisher Scientific). The setting parameters were as follows: enzyme, trypsin; maximum missed cleavage sites, 4; precursor mass tolerance, 10 ppm; fragment mass tolerance, 0.02 Da; static modification, cysteine carbamidomethylation; and dynamic modification, serine, threonine, and tyrosine phosphorylation. The protein identification threshold was set at 1% or less for both peptide and protein FDRs.

### LC-MS/MS Analysis of Biotinylated BSA

A mixture of 10 ng of biotinylated BSA digest and 5 μg of K562 cell digest (Promega) was added to streptavidin beads (10 μl of suspension used; Cat # 21152104010150, Cytiva), washed once with TBST, and mixed for 60 min at room temperature, followed by washing thrice with 1 ml of 50 mM Tris–HCl (pH 8.0) and 500 mM NaCl containing 0.5% SDS and once with 1 ml of Tris-buffered saline. The beads were treated with 50 μl of 8 M guanidine–HCl (pH 1.5) in 0.5 mM biotin or 8 M guanidine–HCl (pH 1.5) in 0.5 mM biotin containing 0.02% LMNG and then mixed for 2 h at room temperature to elute the biotinylated peptides. The eluted peptides were desalted by SDB-STAGE tip (elution: 36% ACN in 0.1% TFA). The dried sample was redissolved in 10 μl of 0.01% DMNG and transferred to a normal vial. The redissolved peptides were directly injected onto a 75 μm × 12 cm nanoLC nano-capillary column (Nikkyo Technos Co, Ltd) at 50 °C and then separated with a 30-min gradient (A = 0.1% FA in water, B = 0.1% FA in 80% ACN) consisting of 0 min 8% B, 26 min 45% B, 29 min 85% B, and 30 min 85% B at a flow rate of 300 nl/min using an UltiMate 3000 RSLCnano LC system. The peptides eluted from the column were analyzed on Q Exactive HF-X using the InSpIon system. MS1 spectra were collected in the range of 450 to 1500 m/z at a 120,000 resolution to set an AGC targets of 3 × 10^6^ and a maximum injection time of 100 ms. The 20 most intense ions with charge states of 2+ to 7+ that exceeded 8.0 × 10^3^ were fragmented by collision induced dissociation with normalized stepped collision energies of 22%, 26%, or 30%. The MS2 spectra were collected in the range of more than 200 m/z at a 60,000 resolution to set an AGC target of 2 × 10^5^ and a maximum injection time of 120 ms. The dynamic exclusion time was set to 30 s. The MS files were searched against the human protein sequence UniProt database (proteome ID UP000005640, 20,591 entries, downloaded on March 7, 2023) and BSA (UniProt Ac P02769) using PEAKS Studio 11 (Bioinformatics Solutions Inc). The parameters were as follows: enzyme, trypsin; maximum number of missed cleavage sites, 2; precursor mass tolerance, 10 ppm; fragment mass tolerance, 0.02 Da; static modification, cysteine carbamidomethylation; and dynamic modification, lysine biotinylation. The protein identification threshold was set at 1% or less for both peptide and protein FDRs.

### Data Analysis

Protein intensities were subjected to a Log2 transformation. Subsequently, proteins were selected from detected valid values in at least 70% of samples within at least one experimental group. Perseus v1.6.15.0 (https://maxquant.net/perseus/) was used for principal component analysis and Pearson’s correlation coefficient heatmap analysis with hierarchical clustering. Missing values were imputed using random numbers drawn from a normal distribution (width, 0.3; downshift, 1.8). For volcano plot, two sample student *t* test (FDR was set to 0.01 and S0 was set to 0.1) were performed using Perseus.

### Experimental Design and Statistical Rationale

**Fig. 1:**
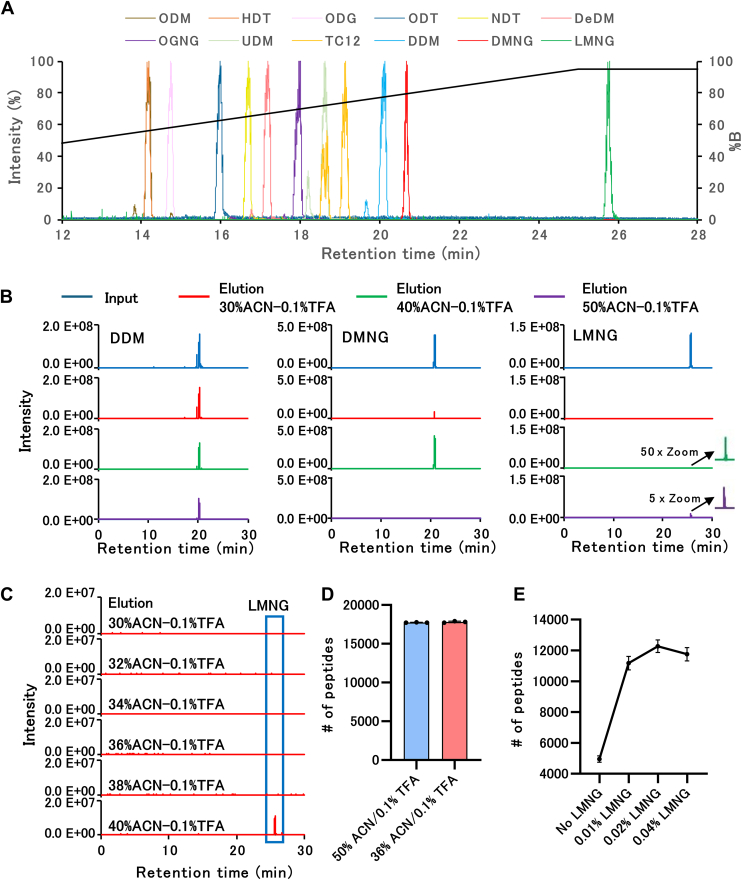
**Development of LMNG removal method by RP-SPE.***A*, MS chromatograms of the monoisotopic mass (single charge) of each surfactant. *B*, elution of LMNG, DMNG, and DDM from the SDB-Stage tip. LMNG, DMNG, and DDM were trapped in SDB-Stage tip and eluted sequentially with 30% ACN in 0.1% TFA, 40% ACN in 0.1% TFA, 50% ACN in 0.1% TFA, and 60% ACN in 0.1% TFA. Subsequently, eluted samples were analyzed by LC-MS. *C*, detailed elution conditions of LMNG from the SDB-Stage tips. LMNG was trapped in SDB-Stage tip and eluted with 30% ACN in 0.1% TFA, 32% ACN in 0.1% TFA, 34% ACN in 0.1% TFA, 36% ACN in 0.1% TFA, 38% ACN in 0.1% TFA, and 40% ACN in 0.1% TFA, in that order, and the eluted samples were analyzed by LC-MS. *D*, comparison of the identification number of peptides eluted from the SDB-Stage tip at 50% ACN in 0.1% TFA and 36% ACN in 0.1% TFA. Ten micrograms of HEK293 cell digest was dried from the tip after elution with each solvent. The dried peptide was then dissolved in 100 μl of 2% ACN in 0.1% TFA, and 1 μl was injected into DDA-LC–MS/MS. *E*, effects of LMNG on peptide recovery. HEK293 cell lysates (100 ng) were analyzed using the SP3 method. Subsequently, 50 mM Tris–HCl pH 8.0 (No LMNG) and 50 mM Tris–HCl pH 8.0 with 0.01% LMNG, 50 mM Tris–HCl pH 8.0 with 0.02% LMNG, and 50 mM Tris–HCl pH 8.0 with 0.04% LMNG were used as digestion solvents. LMNG was removed by SDB-Stage tip (elution: 36% ACN in 0.1% TFA), and peptides were dried. The dried peptide was then dissolved in 8 μl of 2% ACN in 0.1% TFA, and 4 μl was injected into DDA-LC–MS/MS. ACN, acetonitrile; DDA, data-dependent acquisition; DDM, *n*-Dodecyl-β-D-maltoside; DMNG, decyl maltose neopentyl glycol; MS, mass spectrometry; RP-SPE, reversed phase-solid-phase extraction; LMNG, lauryl maltose neopentyl glycol.

**Fig. 2:**
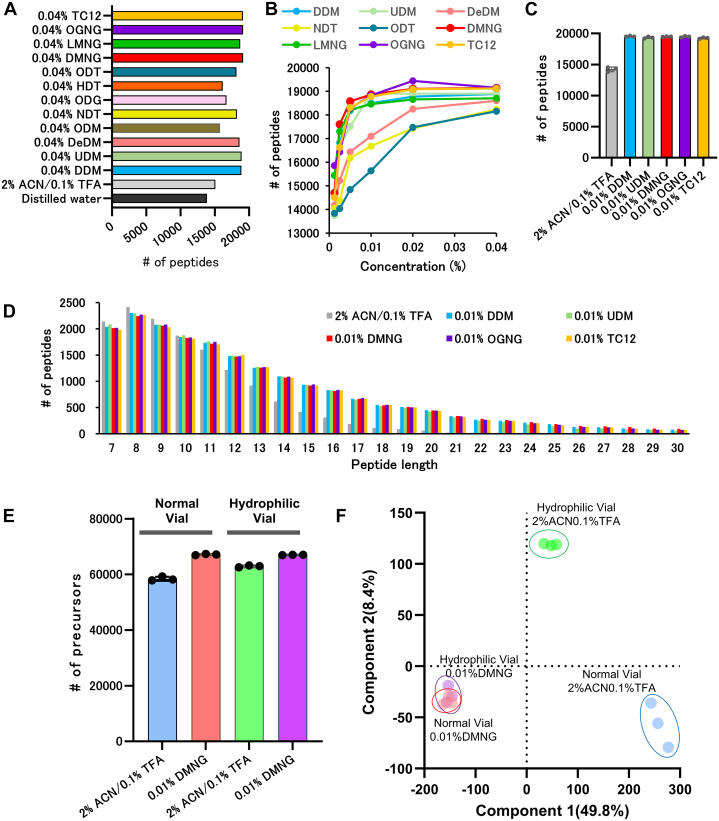
**The recovery of dried K562 cell tryptic peptides by surfactants.***A*, number of peptides identified from dried peptides dissolved in 12 surfactants at 0.04%, 2% ACN in 0.1% TFA, and distilled water. The dried peptide (500 ng) in a 1.5 ml tube was dissolved in 25 μl of each solvent, transferred to a normal PP vial, and 1 μl was injected into DDA-LC–MS/MS (the same treatment was carried out for (*B*) and (*C*)). *B*, number of peptides identified from dried peptides dissolved in nine selected surfactants at 0.00125 to 0.04%. *C*, number of peptides identified from dried peptides dissolved in five selected surfactants at 0.01% and 2% ACN in 0.1% TFA (n = 3). *D*, distribution of amino acid lengths of peptides identified from dried peptides dissolved in each solvent seen in (*C*). *E*, number of precursors identified from dried peptides in normal PP vials or hydrophilic vials dissolved in 2% ACN–0.1% TFA or 0.01% DMNG. The dried peptide (500 ng) in each vial was dissolved in 25 μl of each solvent, and 1 μl was injected into the DIA-LC-MS/MS. *F*, principal component analysis of precursor intensity of the results in (*E*). ACN, acetonitrile; DDA, data-dependent acquisition; DIA, data-independent acquisition; DMNG, decyl maltose neopentyl glycol; MS, mass spectrometry; PP, polypropylene.

**Fig. 3:**
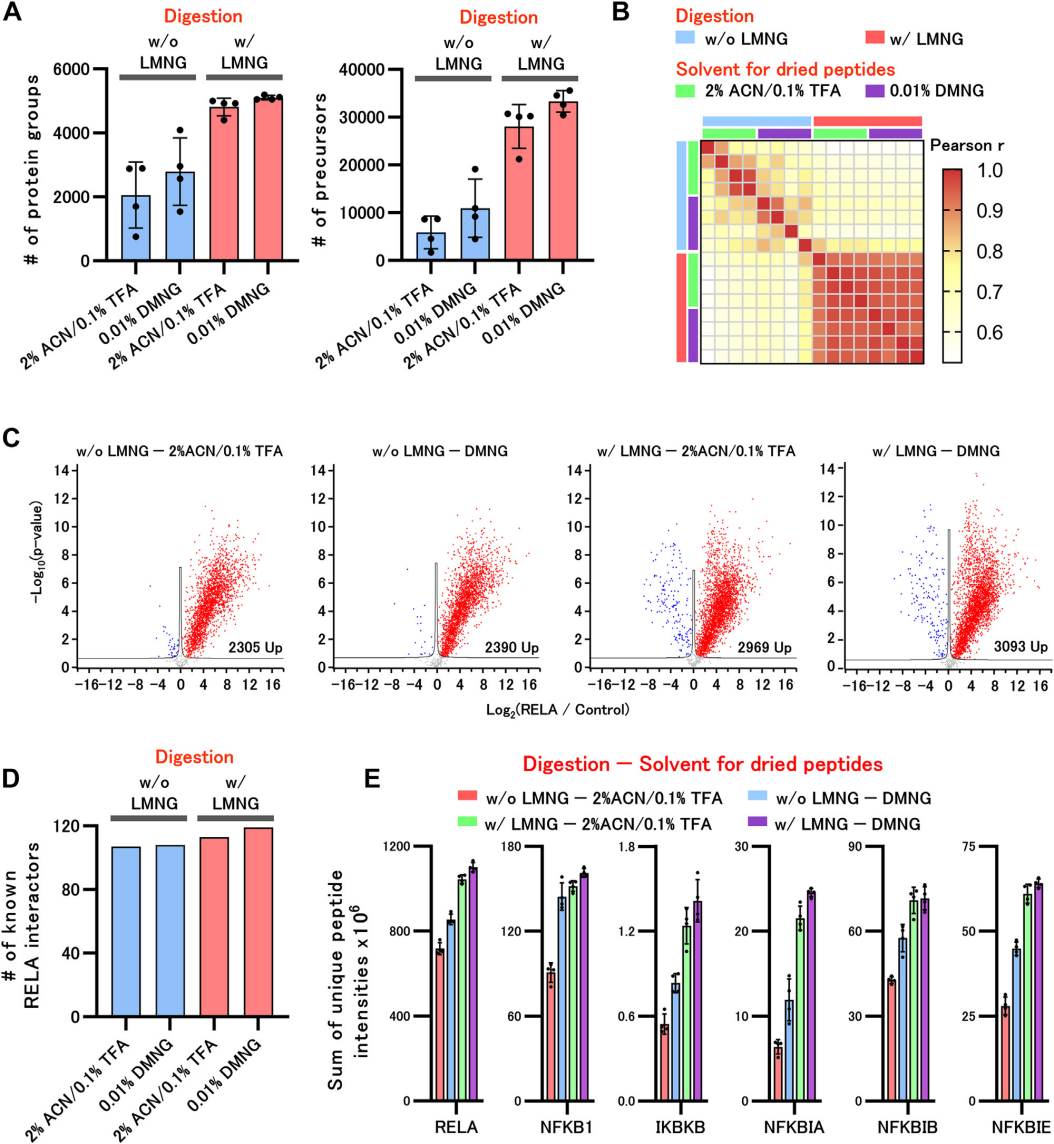
**Combined effect of LASP and DMNG dissolution methods for SP3 and coIP methods.***A*, the number of proteins and precursors identified with and without the addition of LMNG during digestion by the SP3 method and by dissolving dried peptides. Two hundred microlitersof 0.5 ng/μl HEK293 cell lysate was treated using the SP3 method. 50 mM Tris–HCl pH 8.0 with or without 0.02% LMNG was used as the digestion solvent. LMNG was removed using an SDB-Stage tip, and the peptides were dried. The dried peptide was then dissolved in 8 μl of 2% ACN in 0.1% TFA or 0.01% DMNG, and 4 μl was injected into DIA-LC–MS/MS. *B*, Pearson correlation coefficient heatmap with hierarchical clustering of precursor intensity in HEK293 cell proteome analysis. *C*, volcano plot of the protein intensities identified from the coIP-MS using anti-RELA and control antibodies. The coIP was performed using an anti-RELA antibody. Thus, 50 mM Tris–HCl pH 8.0 with or without 0.02% LMNG was used as the digestion solvent. LMNG was removed using an SDB-Stage tip, and the peptides were dried. The dried peptides were then dissolved in 10 μl of 2% ACN in 0.1% TFA or 0.01% DMNG, and 1 μl was injected into DIA-LC–MS/MS. *D*, number of known RELA interactors in the increased proteins extracted from IntAct (https://www.ebi.ac.uk/intact/home). *E*, the sum of the unique peptide intensities of RELA and renowned RELA interactors in the identified proteins. ACN, acetonitrile; coIP, coimmunoprecipitation; coIP-MS, coimmunoprecipitation-mass spectrometry; DIA, data-independent acquisition; DMNG, decyl maltose neopentyl glycol; LASP, (LMNG)-assisted sample preparation; LMNG, lauryl maltose neopentyl glycol; MS, mass spectrometry.

**Fig. 4:**
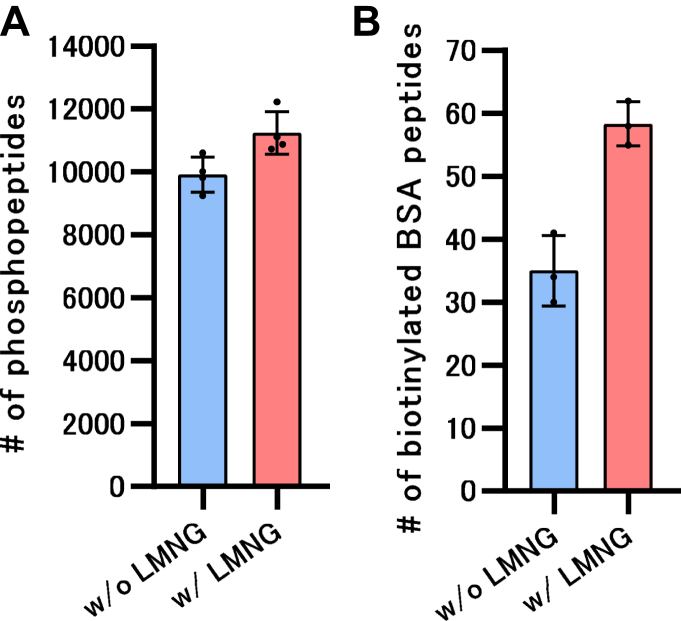
**Effect of LMNG-added elution solvent on affinity purification of peptides.***A*, comparison of phosphopeptides identified with and without LMNG addition during the elution of Fe-NTA magnetic IMAC beads. Phosphopeptides were enriched from 100 μg of HEK293 cell digests. *B*, comparison of biotinylated BSA peptides identified with and without LMNG addition during streptavidin bead elution. Biotinylated BSA peptides were enriched from a mixture of 10 ng of biotinylated BSA digest and 5 μg of K562 cell digest. BSA, bovine serum albumin; IMAC, immobilized metal affinity chromatographic; LMNG, lauryl maltose neopentyl glycol.

**Fig. 5:**
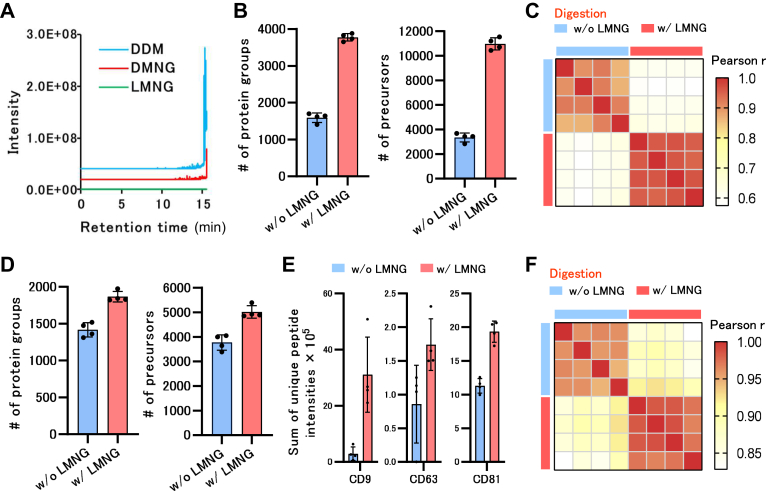
**LASP method using Evosep One system.***A*, elution of LMNG, DMNG, and DDM using EvoSep-One system. LMNG, DMNG, and DDM were trapped in an Evosep Pure tip and set on the Evosep ONE. The Whisper 80 SPD method was used for the LC gradient of Evosep ONE (the same method was used in (*B*) and (*D*)). *B*, the number of proteins and precursors identified with and without LMNG addition during the digestion of HEK293 cell lysates. Subsequently, 200 μl of 0.5 ng/μl HEK293 cell lysate was treated with the SP3 method. A total of 50 mM Tris–HCl pH 8.0 with or without 0.02% LMNG was used as the digestion solvent. *C*, Pearson correlation coefficient heatmap with hierarchical clustering of precursor intensities in HEK293 cell proteome analysis. *D*, number of proteins and precursors identified with and without LMNG addition during serum EVs digestion. EVs enriched with 100 μl of serum were treated using the SP3 method. *E*, sum of the unique peptide intensities of CD9, CD63, and CD81, which are known exosome markers. *F*, Pearson correlation coefficient heatmap with hierarchical clustering of precursor intensities in serum EV proteome analysis. DDM, *n*-Dodecyl-β-D-maltoside; DMNG, decyl maltose neopentyl glycol; EV, extracellular vesicle; LASP, (LMNG)-assisted sample preparation; LMNG, lauryl maltose neopentyl glycol; SPD, samples per day.

**Fig. 6:**
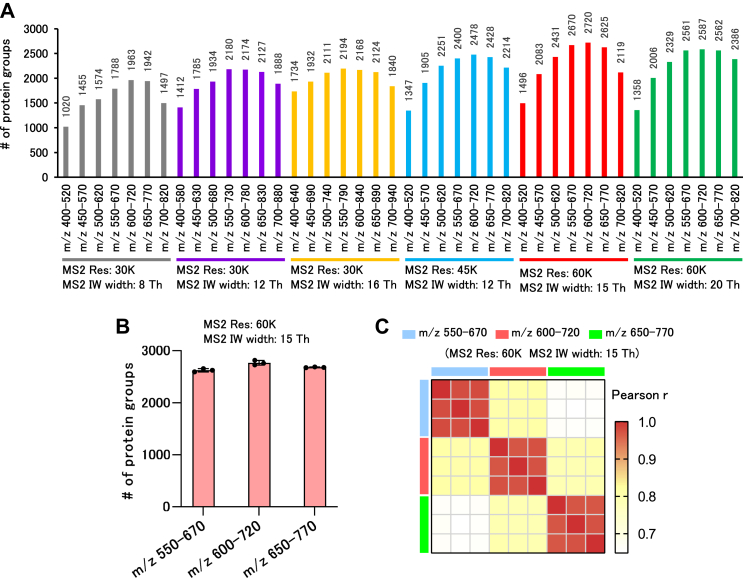
**Investigation of DIA-MS parameters for high-throughput few-cell proteomics analysis.***A*, comparison of protein groups identified using various DIA-MS parameters. One nanogram of the HEK293 cell digest was trapped in an Evosep Pure tip and placed on Evosep ONE. The Whisper 80 SPD method was used for the LC gradient of Evosep ONE (the same method was used in (*B*)). *B*, validation of the method for MS/MS analysis of the m/z 550 to 670, m/z 600 to 720, and m/z 650 to 770 regions with an MS2 resolution of 60 K and an isolation window width of 15 Th. One nanogram of HEK293 cell digest was measured in triplicate for each method. *C*, Pearson’s correlation coefficient heatmap with hierarchical clustering of precursor intensity in the proteome analysis results of 1 ng of HEK293 cell digest. DIA, data-independent acquisition; LC, liquid chromatography; MS, mass spectrometry; SPD, samples per day.

**Fig. 7:**
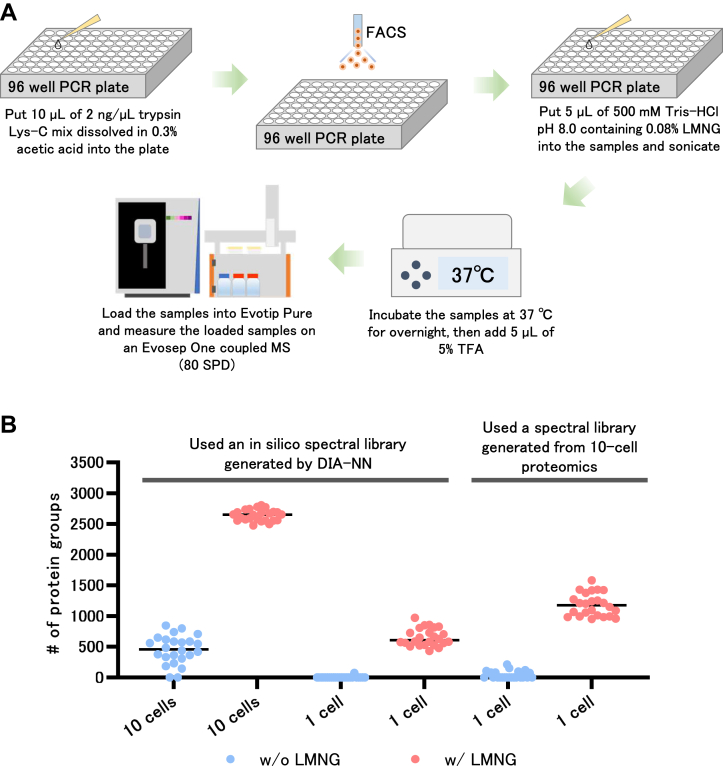
**Label-free SCP by scpLASP method.***A*, workflow of the scpLASP method. We added 10 μl of 2 ng/μl trypsin Lys-C mix in 0.3% acetic acid to a 96-well plate and sorted cells with FACS. Then, we added 5 μl of 500 mM Tris–HCl pH 8.0 containing 0.08% LMNG, followed by sonication and overnight incubation at 37 °C. After that, we added 5 μl of 5% TFA to the digested sample and loaded it into Evotip for analysis on an Evosep One coupled MS with a throughput of 80 SPD. *B*, single and ten cells proteomics with and without LMNG addition during digestion. The DIA-MS data of single and ten cells were first analyzed by the DIA-NN using an *in silico* spectral library. Single-cell DIA-MS data were then analyzed by DIA-NN using a spectral library generated from protein identification information obtained from the proteomics of ten cells. DIA, data-independent acquisition; FACS, fluorescence-activated cell sorting; LASP, (LMNG)-assisted sample preparation; LMNG, lauryl maltose neopentyl glycol; MS, mass spectrometry; SCP, single-cell proteomics; SPD, samples per day.

In this study, the evaluation of LMNG-assisted sample preparation (LASP) efficiency, coIP experiments, enrichment of phosphopeptides and biotinylated BSA, EVs purification, and optimization of DIA for low-input proteome analysis were performed with three (Figs. 1 and 2), four (Fig. 3), four (Fig. 4*A*), three (Fig. 4*B*), four (Fig. 5), and three technical replicates (Fig. 6), respectively. SCP were performed with 24 biological replicates (Fig. 7). These experiments were performed using human-derived samples (HEK293 T cells for the optimization of DIA for low-input proteome analysis, enrichment of phosphopeptides; Freestyle 293 for SCP; plasma for EVs purification; HeLa cells for coIP; K562 cells for comparison of biotinylated BSA). The correlation coefficient for the quantified intensities between LC-MS/MS runs in each group was greater than 0.70. For coIP experiments, we determined that proteins with FDR <0.01 and S0 = 0.1 are statistically significant.

## Results

### Development of a Surfactant Removal Method Using RP-SPE Column for Low-input Proteomics

The surfactants listed in Table 1 (with abbreviations given) were analyzed by LC-MS using a C18 column (Fig. 1*A*; Identifications are listed in supplemental Table S1). The results demonstrate that LMNG had a longer retention time than the other surfactants and a strong affinity for the RP column. The extraction conditions in RP-SPE (STAGE tip) were then examined for LMNG, DMNG, and DDM, which had longer retention times. A commercially available SDB-Stage tip, which is commonly used for desalting digested peptides, was used for RP-SPE. A 50 to 80% ACN solution is generally used as the eluent to ensure that the peptides are eluted. We trapped LMNG, DMNG, and DDM on the SDB-Stage tip and sequentially eluted them with 30% ACN in 0.1% TFA, 40% ACN in 0.1% TFA, 50% ACN in 0.1% TFA, and 60% ACN in 0.1% TFA (Fig. 1*B*). For DDM and DMNG, elution was achieved using 30% ACN in 0.1% TFA, necessitating a lower ACN concentration for their removal. Below 30% ACN, there is a risk of loss of peptides with a strong affinity for the RP carrier. In contrast, LMNG remained uneluted in 30% ACN in 0.1% TFA and only slightly eluted in 40% ACN in 0.1% TFA. Furthermore, a detailed examination of the elution conditions for LMNG revealed that it was not eluted below 38% ACN in 0.1% TFA (Fig. 1*C*). Because the elution of SPE columns changed slightly depending on the room temperature and elution speed, 36% ACN-0.1% TFA was adopted as the condition under which LMNG remained uneluted, with a generous margin. We then investigated whether 36% ACN in 0.1% TFA would be sufficient as an elution solvent for digested peptides (Fig. 1*D*). Importantly, the number of peptides identified remained consistent under the elution conditions of 50% ACN in 0.1% TFA and 36% ACN in 0.1% TFA, and a method was successfully established to remove LMNG without affecting peptide elution on a RP-SPE column. Next, the effect of LMNG on trypsin activity was investigated (supplemental Fig. S1*A*). No significant change in trypsin activity was observed upon the addition of each concentration of LMNG to the digestion buffer. Furthermore, the addition of LMNG to the digestion buffer did not alter the miscutting rate (supplemental Fig. S1*B*) and trypsin and Lys-C activity was not reduced by LMNG. Taking advantage of the easy removal of LMNG, we investigated whether the addition of LMNG to the digestive solution could improve peptide recovery (Fig. 1*E*). We treated 100 ng of HEK293 cell lysate (using 20 μl of 5 ng/μl protein concentration) with the generic SP3 method and confirmed that the addition of LMNG during digestion dramatically increased the number of identified peptides. Notably, the LMNG concentration of 0.02% resulted in the highest number of identified peptides, making it the optimal concentration for the analysis. We have named our LMNG-based method as LASP method. The proteins and peptides used in Figure 1, *D* and *E* are summarized in supplemental Table S1.

### Resolubilization of Dried K562 Cell Tryptic Peptides

To better understand the impact of surfactants on the recovery of dried peptides, we prepared small amounts (500 ng) of dried K562 cell tryptic peptides, which are prone to peptide loss. The dried peptides were dissolved in distilled water, 2% ACN in 0.1% TFA, and 12 surfactants (concentration: 0.04%) for DDA-LC-MS/MS analysis (Fig. 2*A*). The number of identified peptides increased with the use of surfactants. In addition, to identify a surfactant that minimizes MS contamination and dissolves peptides, we tested the effect of dissolving peptides at lower concentrations using nine surfactants that yielded more than 18,000 peptides (Fig. 2*B*). The number of peptides identified in *n*-nonyl-β-D-thiomaltoside, *n*-octyl-β-D-thioglucoside, and *n*-decyl-β-D-maltoside remarkably decreased at lower concentrations. At 0.01% *n*-nonyl-β-D-thiomaltoside, *n*-octyl-β-D-thioglucoside, and *n*-Decyl-β-D-maltoside, the number of identified peptides was considerably reduced. Similar trends were observed for other surfactants, with a noticeable decline starting at 0.005%. Therefore, validation was performed on the top five surfactants (DDM, *n*-undecyl-β-D-maltoside(UDM), DMNG, octyl glucose neopentyl glycol (OGNG), and trehalose C12 (TC12)) with the highest number of peptides identified at 0.01% (Fig. 2*C*). While no significant differences were observed among these selected surfactants, they all proved effective in peptide recovery, even at concentrations as low as 0.01%. Additionally, we investigated the length of peptides identified in each solution and observed that the difference in the number of peptides identified between 2% ACN in 0.1% TFA, and these surfactants, increased as the peptides became longer (Fig. 2*D*). Thus, the use of these surfactants improved the recovery of longer peptides. All five surfactants were effective in dissolving low concentrations of dried peptides; however, we decided to use DMNG, which had the longest retention time that differed the most from the retention time of the peptides. The proteins and peptides used in Figure 2, *A*–*D* are summarized in supplemental Table S2 and S3.

Next, we measured and compared the recovery of 500 ng of dried tryptic peptides in hydrophilic-coated vials and normal PP vials. These peptides were dissolved in 2% ACN in 0.1% TFA or 0.01% DMNG (four-combinations in total) for DIA–LC–MS/MS quantitative analysis. When samples were dissolved in 2% ACN in 0.1% TFA, we identified more precursors in the hydrophilic-coated vials than in the normal PP vials, whereas when samples were dissolved in 0.01% DMNG, recoveries were very similar in the two types of vials (Fig. 2*E*). Regardless of the vial type, more precursors were identified in the dissolution with 0.01% DMNG than in the dissolution with 2% ACN in 0.1% TFA. Principal component analysis based on precursor intensity also showed no differences between vials for DMNG, but there were differences between vials for 2% ACN in 0.1% TFA, with the 2% ACN in 0.1% TFA-hydrophilic vial group being closer to the group dissolved in DMNG (Fig. 2*F*). These results demonstrated improved dried peptide recovery with the use of hydrophilic vials for the dissolution with 2% ACN in 0.1% TFA. In contrast, upon dissolving in 0.01% DMNG, no differences were observed between vial types. Aside from the variation between vials, the use of 0.01% DMNG substantially contributed to the enhanced recovery of peptides. Therefore, the normal vials were sufficient for use with solutions containing 0.01% DMNG. The proteins and precursors used in Figure 2, *E* and *F* are summarized in supplemental Table S4.

### Combined Effect of LASP and DMNG Dissolution Methods

To minimize peptide loss, we tried adding both 0.02% LMNG to the digestion solvent and using 0.01% DMNG to dissolve the dried peptides. In Figure 3, *A* and *B*, 200 μl of 0.5 ng/μl HEK293 cell lysate (protein amount: 100 ng) was used as a starting sample and treated using the SP3 method. The SP3 method faced challenges while processing samples with such low concentrations. The addition of LMNG to the digestion solvent markedly increased the number of identified precursors and proteins, and dissolving dried peptides in DMNG further increased these numbers (Fig. 3*A*). Additionally, in samples without LMNG, the quantitative values of the individual precursors varied remarkably, resulting in low Pearson’s r values (Fig. 3*B*). In contrast, the LMNG samples exhibited higher Pearson’s r values. In the case of low-concentration samples, such as those used in this study, the addition of LMNG not only improved the number of identified proteins and precursors, but also affected the reproducibility of sample preparation. Next, we investigated the effect of adding LMNG during on-bead digestion using coimmunoprecipitation-mass spectrometry (coIP-MS) as well as the effect of dissolving dried peptides in DMNG (Fig. 3, *C*–*E*). In this context, coIP-MS was performed using an anti-RELA and control antibodies. As in the SP3 experiment, the addition of LMNG markedly improved the number of RELA interactor candidates that increased the identification numbers of the precursors and proteins with the anti-RELA antibody; dissolving the dried peptides in DMNG further increased these numbers (Fig. 3*C*). The number of RELA interactors in the IntAct database among the interactor candidates in this study was counted, and the process of adding LMNG to the digestion buffer and dissolving the dried peptides achieved the largest number of interactors (Fig. 3*D*). The recovery of RELA, the antibody target, and its known interactors, NFKB1, IKBKB, NFKBIA, NFKBIB, and NFKBIE, was greatly improved by LMNG and further improved upon lysis with DMNG (Fig. 3*E*). These results confirmed that both the LASP and peptide dissolution methods with DMNG have beneficial effects on coIP-MS. The proteins and precursors used in Figure 3 are summarized in supplemental Table S5. Improvements in the number of identified phosphopeptides and biotinylated peptides were achieved by adding LMNG to the elution solvent in the phosphopeptide and biotinylated peptide enrichment experiments using immobilized metal ion affinity chromatography and streptavidin beads, respectively (Fig. 4, *A* and *B*). Because LMNG can be easily removed by RP-SPE columns, the LASP method is expected to have a wide range of applications, not only for reducing peptide loss during digestion, but also for affinity purification of peptides. The peptides used in Figure 4 are summarized in supplemental Table S6.

### Benefits of the LASP Method in High-throughput Proteomics Using Evosep One

The Evosep One is a well-known LC instrument for high-throughput proteomics and is used worldwide for the proteomic analysis of hundreds to thousands of samples, such as clinical samples. The Evosep One allows for liquid chromatography analysis by trapping the sample in an RP-SPE column [Evotip pure (C18)] and setting it in the Evosep One. For Evosep One, the manufacturer has already prepared an optimized method for peptide analysis that does not allow for fine gradient changes. First, we examined whether LMNG was eluted from the Evosep One. In this study, a high throughput method capable of producing 80 samples per day (SPD) was utilized for all analyses using the Evosep One. LMNG and, controls, DDM and DMNG were also trapped in Evotip Pure and measured by MS after separation using an Evosep One (Fig. 5*A*). Both DDM and DMNG were detected at the end of the retention time, indicating that they were eluted from Evotip Pure, but not LMNG. When Evosep One was used, 100 ng of HEK293 cell lysate at the same low concentration was digested with and without LMNG as shown in Figure 3 and analyzed by LC-MS/MS (Fig. 5, *B* and *C*). More precursors and proteins were identified in LMNG. In addition, even when the EVs were purified from serum samples and analyzed by LC-MS/MS using Evosep One, the addition of LMNG during digestion improved the number of identified precursors and proteins and the recovery of typical exosome markers, such as CD9, CD63, and CD81 (Fig. 5, *D* and *E*). The reproducibility of the quantitative precursor values was further improved upon the addition of LMNG in both experiments (Fig. 5, *C* and *F*). LMNG was also found to be highly compatible with high-throughput proteomic analysis using Evosep One. The proteins and precursors used in Figure 5 are summarized in supplemental Table S7.

### The Challenge of Label-free SCP

SCP requires high throughput to analyze an extremely large number of cells. Therefore, we attempted to establish a system using Evosep One with the 80 SPD method for SCP. For the analytical conditions of MS, we tested the parameters of DIA-MS, thereby allowing for the observation of a large number of proteins, even in trace samples, in the gradient of the 80 SPD method. MS/MS analysis of the m/z 550 to 670, m/z 600 to 720, and m/z 650 to 770 regions with an MS2 resolution of 60 K and an isolation window width of 15 Th in the first screening revealed a higher number of protein identifications (Fig. 6*A*). Furthermore, when the three methods were validated, the method targeting m/z 600 to 720 identified more proteins, albeit only slightly (Fig. 6*B*). All methods showed high protein quantification reproducibility (Fig. 6*C*). Based on these results, we adopted MS/MS at 15 Th intervals for m/z 600 to 720 at an MS2 resolution of 60K as the DIA method for the SCP. The proteins and precursors used in Figure 6 are summarized in supplemental Table S8.

For sample preparation, we established the LASP method for scp (scpLASP), which is 96-well based method and can process multiple samples simultaneously (Fig. 7*A*). The scpLASP method uses a starting volume of 10 μl, making it easy to work with both manual and automatic operations. The scpLASP method was also evaluated with and without the addition of LMNG to the digestion solvent (Fig. 7*B*). Our analyses of HEK293 cells, both 1 and 10 cells showed that the addition of LMNG remarkably increased the number of identified proteins. In scpLASP, protein identification analysis was performed using the *in silico* spectral library generated by DIA-NN, resulting in the identification of a median of 606 and 2650 proteins in 1 and 10 cells, respectively. Furthermore, the results of the 10-cell analysis processed by the scpLASP method were converted to a spectral library. Using that library to analyze the MS data of one cell, a median of 1175 proteins was successfully identified. This was effective in creating a spectral library with bulk samples in advance to perform label-free SCP. To investigate the presence of contaminant proteins in the samples obtained through scpLASP, we searched 151 contaminant proteins reported by Frankenfield *et al.* (21) in the identified protein for scpLASP. Of 1166, 3247, and 1814 identified proteins, 11, 10, and 6 contaminant proteins were detected, respectively. These results indicated that our scpLASP enables the purification of cellular proteins with extremely few contaminants. The proteins and precursors used in Figure 7 are summarized in supplemental Table S9.

## Discussion

We developed the LASP method to suppress peptide loss by LMNG for easy low-input proteomic analysis. Regarding the use of surfactants in low-input proteome analysis, Martin *et al.* (2021) reported reduced peptide loss with the addition of DDM (13, 22). DDM is highly effective because it does not interfere with peptide analysis during LC-MS/MS. However, DDM cannot be easily removed, and when analyzing by LC-MS, depending on the concentration of DDM at the start and the volume of liquid, a large amount of DDM is injected into the LC-MS, increasing the risk of MS contamination (in the case of scp, 2 μl of 0.1% DDM was used for cell lysis). However, if the volume of liquid at the start is markedly reduced, handling becomes difficult, and the method cannot be used universally. From this perspective, LMNG has a considerable advantage in that it can be easily removed with RP-SPE, even if the amount used increases, making it a simple and versatile method. We also used a surfactant to redissolve dried peptides. However, we used a low concentration of 0.01%, and the volume of liquid dissolved was controlled to reduce contamination of the LC-MS by the surfactant. Even if the peptides are dissolved within a few microliters, which make handling more difficult, the impact on the overall protocol is small because it is the final step in the sample preparation process.

To investigate the dissolution of dried peptides, we identified surfactants (DMNG, DDM, UDM, OGNG, and TC12) with high peptide recovery at low concentrations, which can be used for peptide analysis in C18-LC-MS/MS. For DDM, Nie *et al.* (2022) (12) reported improved peptide recovery similar to our results. The effects of DMNG, UDM, OGNG, and TC12 were, for the first time, investigated in our study. Although LMNG was effective in recovering dried peptides, the affinity of LMNG for C18 was so strong that the risk of carryover was high and LMNG was not suitable for continuous analysis. However, LMNG may be suitable for protein lysis in top-down proteomic analyses. Because the C4 and C8 columns are used in top-down proteomic analysis, the risk of LMNG carryover is lower than that for C18. Moreover, in RP-HPLC, proteins tend to elute at later retention times than peptides, which raises the concern of potential overlap between protein elution times and surfactants as DMNG, DDM, UDM, OGNG, and TC12, commonly used in peptide analysis. Therefore, the use of surfactants with extremely late elution times, such as LMNG, offers an advantage in this context. The late retention time characteristic of LMNG has both advantages and disadvantages, but we believe that it can be widely applied if it is exploited. After 2 months of measuring various digests dissolved in DMNG for MS contamination, there was no significant change in the number of proteins identified in the HEK293 digests, which were regularly measured for MS quality control. The use of low concentrations of DMNG (0.01%) did not present significant problems with LC-MS. Hydrophilic coating of the LC vials was not necessary. As long as the peptides were dissolved in DMNG, the normal PP vials were sufficient. Hydrophilic-coated vials and tubes are expensive, whereas dissolution with surfactants such as DMNG is inexpensive because high numbers of peptides can be recovered even with normal PP vials and tubes.

In SCP, hundreds to thousands of analyses are necessary, requiring high-throughput analysis of samples that are orders of magnitude larger than those used in the past. Therefore, the risk of MS contamination should be eliminated as much as possible. The combined LASP and Evosep system is a highly sensitive throughput method for reducing peptide loss and MS contamination. Although SCP sample preparation is generally performed with a small volume of liquid to reduce protein and peptide loss, we established the scpLASP method, which does not require delicate handling of the sample. A typical SCP requires careful setting up of the wells such that the sorted cells from the FACS are sent to the bottom of the wells, and the cells are placed in a few microliters of solvent placed at the bottom of the wells (13, 23, 24). The scpLASP method encompassed 10 μl in the bottom of the well to allow for easy collection of sorted cells. The presence of 10 μl of liquid from the start lowered the risk of the sample drying out, facilitated the addition of reagents and agitation, and simplified the overall handling of the sample preparation. A higher liquid volume did not present a major problem in our method because the surfactant added to reduce protein and peptide loss could be removed at the end of the Evotip Pure. In addition, 10 μl of 2 ng/μl trypsin Lys-C in 0.3% acetic acid was dispensed in 96 PCR well plates and stocked at −80 °C for less preparation during FACS. Enzymatic digestion was initiated by adding 500 mM Tris–HCl pH 8.0 with 0.08% LMNG to the sorted cell samples, and peptide loss was also reduced.

Thus, our scpLASP method is simplified, and we believe that it is an effective method not only for SCP, but also for spatial proteomics. We also believe that the scpLASP method will become the standard method for ultra-low-input sample preparation, such as in SCP and spatial proteomics, owing to its simplicity and low risk of MS contamination.

Our SCP results showed that the combination of a simple pretreatment and 80 SPD LC-MS/MS allowed for the identification and quantification of a median of 1175 proteins from a single HEK293 cell without enhancing the observed protein count by match between runs, indicating that our total analysis system was also superior. However, there are more optimal conditions for LC column and MS equipment. For LC column, micropillar array columns suitable for low-input samples have been developed and are commercially available, albeit expensive. Compared to the 50 cm packed bed column, the 50 cm pillar column identified more than 1.5 times the number of proteins in the LC-MS/MS analysis of 10 ng of the HeLa digest (25). In label-free SCP, although the LC-MS/MS throughput was 40 SPD, an average of 1543 and 1404 proteins were quantified in a single HeLa cell and a single K562 cell, respectively, using a 5.5 cm micro-pillar array column (26), and approximately 2000 proteins were quantified from a single HEK293 cell using a 50 cm micropillar array column (24). MS data acquisition and data analysis methods were developed for SCP in these studies; however, the benefits of using micropillar array columns were considered significant. Furthermore, in June 2023, Orbitrap Astral (Thermo Fisher Scientific) was released as an ultrasensitive MS, and surprisingly, upon using the Orbitrap Astral with a 50 cm micropillar array column, approximately 2500 proteins were identified from a single HEK293 cell at an LC-MS/MS throughput of 80 SPD (27). Bruker Daltonics Inc simultaneously released the timsTOF Ultra, an ultrasensitive MS that represents a significant advancement for SCP. We believe that by combining these new techniques with the scpLASP method, a high-level SCP can be easily achieved.

## Data Availability

Mass spectrometry proteomics data were deposited in the ProteomeXchange Consortium via the jPOST partner repository (28) with the dataset identifiers PXD044605 and PXD048100 for ProteomeXchange, and JPST002281 and JPST002431 for jPOST (URL: https://repository.jpostdb.org/preview/522211216651109d92eac1) (URL https://repository.jpostdb.org/preview/8215915656588b9a4e5f93).

## Supplemental data

This article contains supplemental data.

## Conflict of interest

The authors declare no competing interests.
