# Supplementary material for: Universal Pretreatment Development for Low-input Proteomics Using Lauryl Maltose Neopentyl Glycol
Source: Mol Cell Proteomics. 2024 Mar 4;23(4):100745. doi: 10.1016/j.mcpro.2024.100745 (PMC10999711; doi:10.1016/j.mcpro.2024.100745)
Supplement: Supplemental Figures [file mmc1.pdf]

Universal pretreatment development for low-input proteomics using lauryl maltose neopentyl glycol

Running Title: LMNG-Assisted Sample Preparation for SCP

Ryo Konno[1 §], Masaki Ishikawa[1 §], Daisuke Nakajima[1 §], Yusuke Endo[2], Osamu Ohara[1], Yusuke Kawashima[1,3*]

[1]Department of Applied Genomics, Kazusa DNA Research Institute, Kisarazu, Chiba 292-0818, Japan.

[2]Department of Frontier Research and Development, Kazusa DNA Research Institute, Kisarazu, Chiba 292-0818, Japan.

[3]Graduate school of science, kitasato university, Sagamihara, Kanagawa 252-0373, Japan.

[§] These authors contributed equally

*Correspondence: Email: ykawashi@kazusa.or.jp; Tel: +81-438-52-3580, Fax: +81-438-52-3501

Supplemental Figure

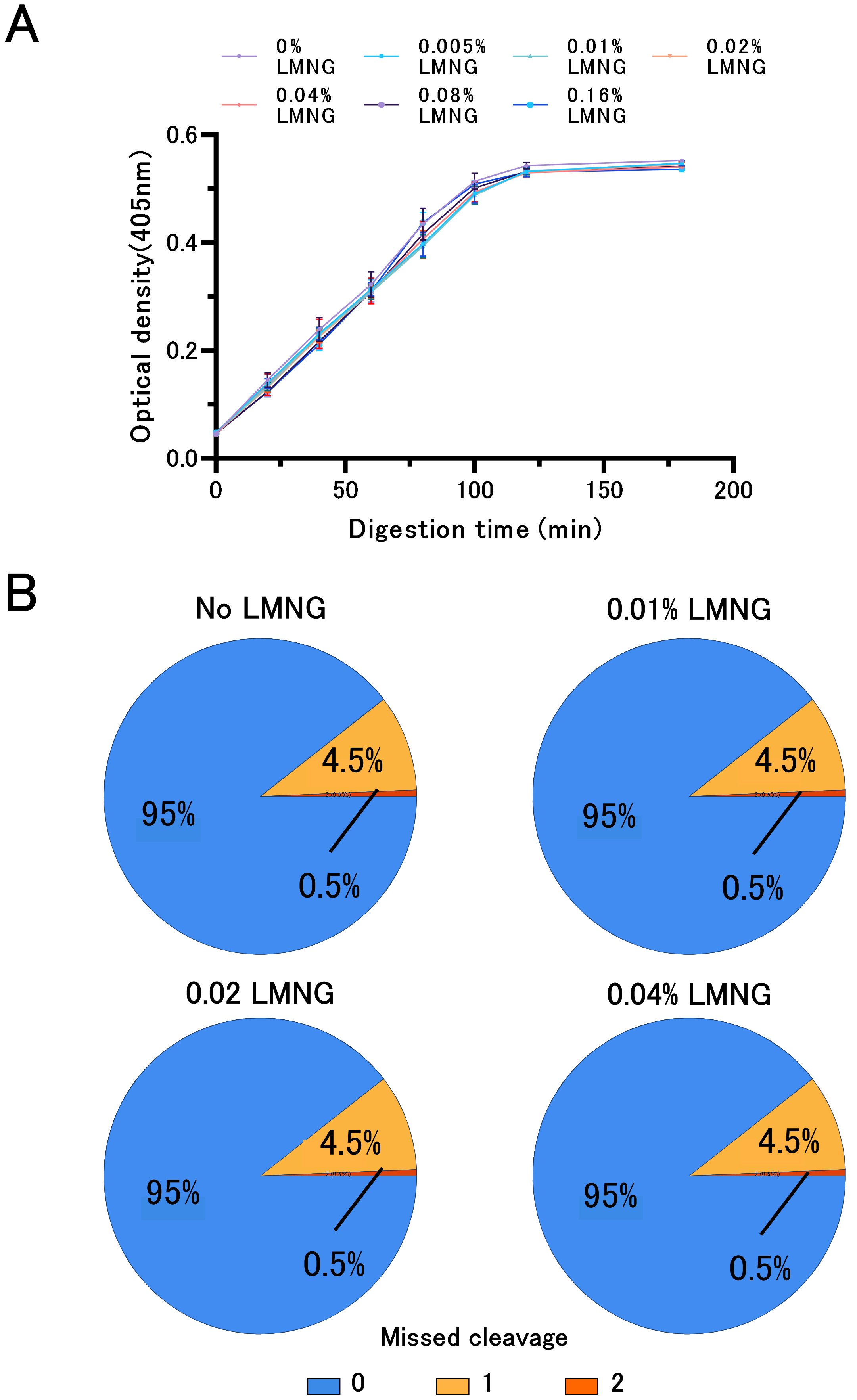

Fig. S1 Effects of LMNG on trypsin activity.

A) The trypsin activity was investigated for the digestion buffer without LMNG and with 0.005%, 0.01%, 0.02%, 0.04%, 0.08% and 0.16% LMNG. The solutions were added 6.25 mU of trypsin, and the trypsin activity was then measured with absorbance at 405 nm.

B) The missed cleavage rate was calculated for trypsin digestion buffer without LMNG and with 0.01%, 0.02%, and 0.04% LMNG. Trypsin Lys-C mix was added at 1μg for 20μg of HEK293 proteins.
